# Emerging roles of telomeric chromatin alterations in cancer

**DOI:** 10.1186/s13046-019-1030-5

**Published:** 2019-01-17

**Authors:** Stefano Cacchione, Annamaria Biroccio, Angela Rizzo

**Affiliations:** 1 Department of Biology and Biotechnology “Charles Darwin”, Sapienza University of Roma, Piazzale Aldo Moro 5, 00185 Rome, Italy; 2 Oncogenomic and Epigenetic Unit, IRCCS-Regina Elena National Cancer Institute, Via Elio Chianesi 53, 00144 Rome, Italy

**Keywords:** Telomere, Cancer, Chromatin, Epigenetics, Sirtuins, Heterochromatin, ALT

## Abstract

Telomeres, the nucleoprotein structures that cap the ends of eukaryotic chromosomes, play important and multiple roles in tumorigenesis. Functional telomeres need the establishment of a protective chromatin structure based on the interplay between the specific complex named shelterin and a tight nucleosomal organization. Telomere shortening in duplicating somatic cells leads eventually to the destabilization of the telomere capping structure and to the activation of a DNA damage response (DDR) signaling. The final outcome of this process is cell replicative senescence, which constitute a protective barrier against unlimited proliferation. Cells that can bypass senescence checkpoint continue to divide until a second replicative checkpoint, crisis, characterized by chromosome fusions and rearrangements leading to massive cell death by apoptosis. During crisis telomere dysfunctions can either inhibit cell replication or favor tumorigenesis by the accumulation of chromosomal rearrangements and neoplastic mutations. The acquirement of a telomere maintenance mechanism allows fixing the aberrant phenotype, and gives the neoplastic cell unlimited replicative potential, one of the main hallmarks of cancer.

Despite the crucial role that telomeres play in cancer development, little is known about the epigenetic alterations of telomeric chromatin that affect telomere protection and are associated with tumorigenesis. Here we discuss the current knowledge on the role of telomeric chromatin in neoplastic transformation, with a particular focus on H3.3 mutations in alternative lengthening of telomeres (ALT) cancers and sirtuin deacetylases dysfunctions.

## Background

The presence of a mechanism to maintain telomeres - the nucleoprotein structures at the end of human chromosomes - is essential to allow the indefinite proliferation capacity of cancer cells. Due to the inability of DNA polymerases to completely replicate the ends of linear DNA molecules, known as the end-replication problem, eukaryotic chromosomes shorten at each duplication cycle. At birth, human telomeres typically consist of 10–15 kilobases (kb) of double-stranded TTAGGG repeats ending in a 50–400 nt long 3′-extension of the G-rich strand. Linear ends need also to be protected from being recognized as DNA breaks and being incorrectly repaired by fusion with other chromosomes. End-protection is assured by a six-protein complex, shelterin, which binds and cap telomeres (see ref. [1] for an extensive and complete review). Human shelterin is anchored to double-stranded telomeric DNA by the binding of TRF1 and TRF2; TIN2 connects TRF1, TRF2, and TPP1, which in turn binds POT1, which recognizes the single-stranded protrusion. The sixth protein, Rap1, interacts with TRF2. Shelterin caps human telomeres by forming t-loops, lariat-like structures in which the single-stranded 3′-overhang invades the upstream double-stranded telomeric DNA [2].

**Fig. 1:**
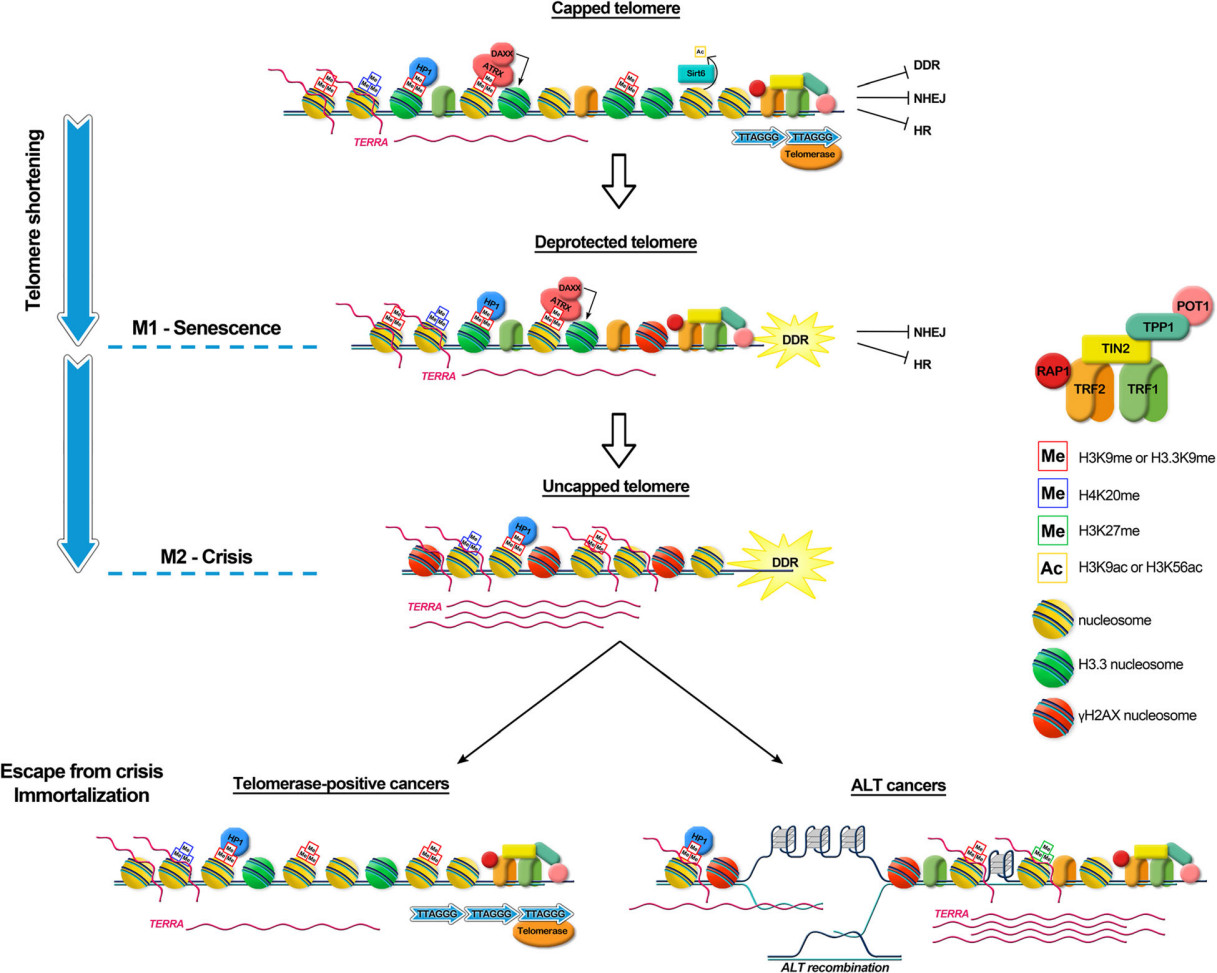
Schematic representation of different healthy and pathological telomeric states. The figure shows the changes of the telomere structure from a capped telomere to neoplastic transformations (from top to bottom). The t-loop structure, as several proteins that play a role at telomeres, are not shown for sake of clarity. Capped telomere: the shelterin complex protect telomeres from DNA damage response and from DNA repair pathways. Telomeric chromatin is maintained in a hypoacetylated, heterochromatic form by the action of the deacetylase SIRT6, ATRX promotes the incorporation of histone H3.3 and resolves G-quadruplex structures and R-loops. Deprotected telomere: telomere shortening leads to the disruption of the closed conformation and to the activation of DDR signaling. Cells undergo a growth arrest named replicative senescence or M1 (mortality stage 1). There is still enough shelterin proteins to block non-homologous end joining (NHEJ) and homologous recombination (HR) pathways. Uncapped state: Inactivation of growth arrest checkpoint (p53) allows cells to bypass M1. This leads to excessive telomere shortening, until cells reach a state termed crisis (or M2) characterized by extensive cell death. Telomeres are fully uncapped, loss of shelterin leads to activation of DNA repair pathways, resulting in telomeric fusions. Rarely, premalignant cells escape from crisis acquiring a telomere maintenance mechanism that permits unlimited proliferation. In most cases, by reactivating telomerase (on the left); 10–15% tumors develop an alternative mechanism named ALT (on the right), characterized by high TERRA levels, R-loops, DDR, and maintenance of telomere length by homologous recombination

Telomere length maintenance and telomere protection are interdependent, since telomere shortening induces telomere deprotection and chromosome instability (see Fig. 1 for a schematic description). In most eukaryotes, end-erosion is counteracted by the action of the ribonucleoproteic enzyme telomerase, which adds short repeats to the 3′ ends of chromosomes, the telomeres [3]. In humans, telomerase is active only in germinal and in stem cells. Consequently, most human somatic cells undergo programmed telomere shortening [4]. When telomere attrition is such to determine a loss of telomere protection, the activation of DDR at chromosome ends causes the arrest of cell proliferation by inducing senescence or apoptosis (Mortality stage 1, M1) [5]. This telomere proliferation barrier has long been recognized as a tumor suppressor mechanism [6]. However, if mounting telomere dysfunction is coupled to the impairment of pathways necessary for cell cycle arrest, a transient event of telomere crisis (Mortality stage 2, M2) occurs, leading to extensive genome instability [7]. At this stage, dysfunctional telomeres do not anymore direct cells towards senescence but instead represent a source of genomic instability that favors tumorigenesis [8, 9]. To escape from crisis, incipient cancer cells require the re-activation of telomerase or the establishment of a telomerase-independent maintenance mechanism named ALT, based on homologous recombination (HR) among telomeres [10]. Telomere maintenance confers unlimited proliferative potential to pre-neoplastic cells, allowing also the stabilization of a heavily rearranged genome that has acquired new and potentially tumorigenic genetic mutations. In most cancers immortalization derives from telomerase reactivation [11]; the remaining 10–15% of tumors are telomerase-negative and utilize the ALT mechanism of telomere maintenance [12, 13]. Reactivation of telomere maintenance programs also enables the transmission of abnormal chromosomal structures (i.e., amplifications, deletions, translocations, inversions) that arise as a result of iterative chromosomal breakage-fusion bridge cycles [7].

Given the crucial role telomeres play in cancer development, studying the mechanisms of telomere protection and the changes in telomere structure during tumorigenesis is essential to understand the biology of cancer and develop effective therapeutic strategies. Here we review the modifications of the structure and the epigenetic state of telomere chromatin that occur upon cancer establishment, with a particular emphasis on the role of H3.3 mutations in pediatric ALT tumors and on telomere dysfunctions derived by altered expression of sirtuin deacetylases.

## Structure of human telomeric chromatin

Shelterin complexes bind telomeric DNA as independent units [14], in a chromatin environment characterized by an atypical nucleosomal organization (see ref. [15] for a review on the argument). Telomeric nucleosomes in human cells have a repeat length of 160 bp, about 40 bp shorter than in the rest of chromatin [16]. Moreover, in vitro studies showed that telomeric nucleosomes are less stable than average nucleosomes [17] and can slide along telomeric DNA [18]. The telomeric nucleosomal organization seems to persist until the very end of the chromosome [19], limiting and affecting shelterin access to telomeric DNA. Furthermore, studies on mouse cell lines show that shelterin removal has no effect on the nucleosomal organization at telomeres [19, 20]. These results suggest that shelterin and the other proteins involved in telomere function have to interplay with a stable nucleosomal scaffold and not with naked DNA. Kinetic studies showed that nucleosomes have a very low turnover [21], whereas the proteins that compose the shelterin complex have a very rapid exchange at telomeres [22], mainly by 3D diffusive search of telomeric sequences [14]. Telomerase also accesses telomeres in S-phase with high frequency [23]. In vitro studies showed that the presence of nucleosomes modulates binding of TRF1 and TRF2 to telomeric double-stranded repeats [24, 25], indicating that TRF1 has a much higher affinity than TRF2 both to nucleosomal binding sites and to linker DNA. Other studies suggest that TRF2 can induce compaction of telomeric chromatin [26] and that TRF2 overexpression can alter nucleosomal spacing in a cancer cell line [27].

Whether nucleosomal organization plays a role in human telomere protection is still an open matter. Recently, it was proposed that access of DDR factors to deprotected telomeres might depend on decompaction of telomeric chromatin upon loss of TRF1 and TRF2 [28]. Contrary to these findings, other recent works suggest that DDR response at telomeres as a consequence of shelterin depletion does not significantly change telomere compaction and accessibility [29, 30]. Mammalian telomeric chromatin is generally considered heterochromatic [31–33], enriched in heterochromatic marks such as trimethylation of Lys9 of histone H3 (H3K9me3) and Lys20 of histone H4 (H4K20me3) (Fig. 1). However, this concept is based mainly on data obtained on mouse telomeres [34]. The epigenetic state at human telomeres is less typically heterochromatic [15]. ChIP and ChIP-seq experiments show unexpected low levels of H3K9me3 at telomeres in human fibroblasts [35], in human CD4C T-cells [36], and in nine human cell lines of different origin [37, 38]. Clear heterochromatic marks such as H3K9me3 and DNA hypermethylation characterize instead subtelomeric regions [36, 38]. However, other direct and indirect evidences support the importance of a heterochromatic state for healthy human telomeres. Specifically, hypoacetylation of lysines 9 and 56 of histone H3 – a typical heterochromatic pattern - is essential for a correct telomere capping [39, 40]. In addition, the heterochromatin protein HP1-γ interacts with the shelterin protein TIN2 and is required for telomere cohesion during S-phase [41]. Another peculiar feature of telomeric chromatin is the enrichment for the H3 histone variant H3.3 [42]. H3.3 is expressed throughout the cell cycle by two genes, *H3F3A* and *H3F3B*, located on chromosomes 1 and 17, respectively. Enrichment for H3.3 was first found within actively transcribed genes, via a replication-independent deposition mechanism catalyzed by the histone chaperone Histone Regulator A (HIRA) [42, 43]. More recent studies showed that histone H3.3 is also incorporated in telomeres by a complex comprising the α-thalassemia/mental retardation syndrome X-linked protein (ATRX) in cooperation with the histone chaperone death domain-associated protein 6 (DAXX) [42, 44, 45], also involved in H3.3 deposition at imprinted genes and interstitial heterochromatic sites [46]. The HIRA complex and the ATRX-DAXX complex control replication-independent deposition of H3.3 at distinct sites on the genome [42, 45]. These specific deposition mechanisms indicate that H3.3 has multiple and distinct functions. The role played by H3.3 in telomere homeostasis is still unknown.

However, heterochromatin formation does not impede that telomeres are actively transcribed to generate long non-coding UUAGGG-repeated RNAs named TERRA (telomeric repeat–containing RNA) [47, 48]. Even if the mechanisms of TERRA functions have to be fully elucidated, it is now commonly recognized that TERRAs are implicated in important telomere functions [49], including telomere homeostasis [50], and telomere protection [51, 52]. Importantly, several evidences show that TERRA interacts with TRF1 and TRF2 and is involved in heterochromatin formation [53]. Moreover, it has been shown that TERRA interacts with heterochromatin protein 1 (HP1) and with telomeric chromatin containing H3K9me3 [53–55]. Upon TRF2 depletion, TERRA transcription is upregulated and TERRA interacts with the histone methyltransferase SUV39H1, promoting methylation of histone H3K9 [56].

## Shelterin alterations and cancer

Several mutations and/or altered expression in shelterin components at telomeres have been described in cancer, but how these components are regulated during different stages of cancer development is not well understood. Patients with early-stage chronic lymphocytic leukemia (CLL) have an increased frequency of dysfunctional telomeres and telomere-to-telomere fusions are observed in advanced stages of the disease [57, 58]. In agreement with a role of telomere-dysfunction in CLL, reduced expression levels of TRF1, RAP1 and POT1 [59], as well as TIN2 and TPP1 [58] have been detected. Furthermore, somatic mutations in POT1 account for 5% of CLL cases [60]. Of note, in addition to leukemia, mutations in POT1 or RAP1 have been found to be mainly associated with familial melanoma [61, 62], familial glioma [63], Li-Fraumeni-like syndrome [64], mantle cell lymphoma [65] and parathyroid adenoma [66]. The malignant-predisposing mutations in the POT1 gene, which alter the ability of the shelterin protein to bind to single-stranded telomeric DNA, lead to the fusion of sister telomeres and are associated to increased telomere length, owing to the loss of POT1-mediated inhibition of telomerase [67]. These findings provide novel insights into how genomic instability induced by dysfunctional telomeres contributes to tumorigenesis. On one side, POT1 inhibition may result in defective telomere replication caused by impaired CST (CTC1-STN1-TEN1) function at telomeres, thus promoting a telomere-driven genome instability [68]. On the other, the presence of longer telomeres may reduce the tumor-suppressive effects of telomere attrition as consequence of a delayed senescence onset in precancerous dividing cells. Additionally, POT1 and RAP1 expression appeared deregulated in hepatocellular carcinoma (HCC) [69]. Finally, TRF1 and TRF2 were reported to be up-regulated in several cancer types such as lung, gastric, breast, colon and renal tumors [70–74]. The role of the shelterin gene mutations in cancer rely mainly on the perturbation of their telomere-related activities impacting on telomere integrity. However, the putative roles of TRF2 in tumorigenesis, as well as of RAP1, have been ascribed also to extra-telomeric functions. By combining chromatin immunoprecipitation with high-throughput DNA sequencing (ChIP-Seq), it has been shown that TRF2 and RAP1 occupy both telomeric and extratelomeric TTAGGG repeats throughout the human genome, referred to as interstitial telomeric sequences (ITSs), where they can affect gene transcription [75–77]. Specifically, RAP1 associates to both subtelomeric related genes and genes linked to metabolic regulation, cell adhesion, and cancer [75]. Additionally, RAP1 can translocate to the cytoplasm, where it acts as a modulator of the NF-kB signaling pathway by interacting with IKK complex. The RAP1-IKK interaction is required for the phosphorylation of the p65 subunit of NF-kB, enabling it to perform gene transcriptional activation [78]. By binding ITSs, TRF2  modulates the HS3ST4 gene, encoding heparan sulfate (glucosamine) 3-O-sulphotransferase 4, which is involved in regulating NK cell recruitment/activation at the tumor site with an impact on tumor take/growth [79]. By localizing directly to specific promoter regions, TRF2 regulates the expression of the platelet-derived growth factor receptor-β (PDGFRβ; [80]), thus promoting angiogenesis; furthermore, TRF2 represses the cyclin-dependent kinase p21 (CDKN1A/CIP1/WAF1) through the REST-LSD1 repressor complex recruitment [81].

Collectively, these findings implicate that an altered expression of shelterin genes, besides impacting on telomere homeostasis, may have substantial consequences on extra-telomeric loci, thus integrating telomeric chromatin alterations with aberrant gene transcription profiles. Consistently, looping of telomeres to interstitial sites, referred to as interstitial t-loops, mediated through TRF2 and lamin associations has been reported [82]. More recently, Mukherjee et al. [83] have shown that binding of TRF2 at promoters about 60 Mbp from chromosome ends depends on telomere length in human cells. Promoter TRF2 occupancy was affected in cells with elongated telomeres producing an altered TRF2-mediated transcription of distal genes.

## Epigenetic alterations of telomeric chromatin in cancer

It is still not clear whether telomerase-positive cancer cells are characterized by a specific epigenetic pattern. Roles for epigenetic regulation of telomere maintenance have been reported in mouse. Knockout of various chromatin remodeling factors (CRFs), such as histone methyltransferases SUV39H1/2, SUV4-20H1/2 result in defective telomere function, aberrantly increased telomere length, and chromosomal instability (see ref. [84] for a review). In humans, SIRT1 and SIRT6, both members of the mammalian sirtuin family of Nad + −dependent histone deacetylases, are among the most extensively studied CRFs interacting with telomere-repeats implicated in telomere integrity [39, 85–90]. Specific epigenetic changes have been associated with ALT cancers, such as the increase of TERRA transcription and enrichment of heterochromatic marks [52]. Importantly, high frequency of H3.3 point mutations and/or ATRX/DAXX mutations have been associated with pediatric cancers [91–94] and with the establishment of a ALT mechanism of telomere maintenance [95].

### Sirtuins

Deacetylation activity of SIRT1 is directed against both histone and non-histone targets, implying the involvement of SIRT1 in several cellular functions including energy metabolism, cellular stress resistance, genomic stability, aging and tumorigenesis (reviewed in [96]). SIRT1 was firstly demonstrated to be recruited to telomeres in murine pluripotent stem cells (iPSCs) and to positively regulate telomere length in both mouse embryonic fibroblasts and tissues [86]. Chen et al. [88] have reported that SIRT1-silencing causes nuclear abnormalities, telomere dysfunction induced foci and induced cellular senescence in HCC cells by inhibiting the shelterin TPP1 expression. Indeed, up-regulated expression of TPP1 in SIRT1-depleted HCC cells improved cellular senescence, strongly suggesting that TPP1 was closely involved in the SIRT1-mediated anti-senescence effects in HCC cells [88]. Another study showed that SIRT1 is necessary for telomere elongation after reprogramming of murine and human somatic cells, and it is required to maintain genomic stability, telomeric transcription and remodeling of telomeric chromatin [90].

SIRT6 is a complex enzyme with multiple substrates and catalytic activities, as deacetylation of both histones and non-histone proteins, deacetylation of long-chain fatty acyl groups and mono-ADP-ribosylation activity [97]. At chromatin level, SIRT6 deacetylates the histone H3 on acetylated K9, K56 [39, 98] and the more recently identified K18 and K27 residues [98–100], causing the repression of many genes differently involved in inflammation, aging, genome stability, metabolic pathways and telomere integrity [101, 102]. Upon DNA damage, SIRT6 is recruited to double strand breaks (DSBs) ensuring the proper activation of downstream DDR factors leading to an efficient repair [87]. In 2008, Michishita et al. [39] showed that SIRT6-mediated deacetylation of histone H3 on acetylated lysine 9 (H3K9ac) modulated telomeric chromatin structure. Specifically, SIRT6 can localize to the telomeric chromatin and its loss leads to the dysfunction of telomeres resembling a phenotype of telomere abnormality similar to that of Werner syndrome [39, 40, 98], with chromosome end fusions and cellular senescence. The Werner syndrome ATP-dependent helicase (WRN) is a well-known RecQ-like helicase that plays a major role in genome stability, particularly during DNA replication and telomere metabolism [103]. In detail, SIRT6 deacetylates H3K9 at telomeric chromatin and is required for the stable association of WRN. Additionally, SIRT6 is required for proper replication of telomeres by deacetylating H3K9 and H3K56 during S-phase [40]. Thus, depletion of SIRT6 from human cells resulted in abnormal telomere structures and stochastic replication-associated telomere sequence loss, ultimately leading to chromosomal end-to-end fusions and consequent genomic instability [87]. A very recent paper attributes to SIRT6 the ability to facilitate directional telomere movement upon oxidative damage by recruiting SNF2H (an ATP-dependent chromatin-remodeling factor) with resulting local chromatin decondensation at telomeres [104]. Another important function of SIRT6 at telomeres is the ability to prevent impaired telomere position effect (TPE), the epigenetic silencing of telomere-proximal genes [87]. Indeed, RNAi-mediated depletion of SIRT6 abrogated silencing of both an integrated telomeric transgene and an endogenous telomere-proximal gene. Moreover, enhanced telomeric silencing in response to telomere elongation is associated with increased repressive chromatin marks, and this heterochromatic milieu is lost in SIRT6-deficient cells. These findings may be relevant in suggesting an additional mechanism by which telomeric chromatin may contribute to tumorigenesis. Since aberrant expression of silent chromatin has been increasingly recognized to have a role in cancer [105], it would be interesting to understand if telomere erosion, as well as SIRT6 inhibition —and consequent de-repression of telomere-proximal genes—may impact on cancer-related changes in gene expression [106, 107]. Interestingly, in line with this notion, recently published data suggest that histone modifications typical of chromatin compaction (H3K27me3) or access (H3K4me1 and H3K4me3) to regulatory factors, at sites distant from telomere ends depend on telomere length [83]. Moreover, loss of silencing factors from shortening and/or dysfunctional telomeres might lead to a relocalization of these factors from chromosome ends to other genomic loci, triggering aberrant silencing of non-telomeric genes [108].

The role of SIRT6 in cancer is controversial. In some tumors, high levels of SIRT6 are associated with poorer outcomes [109, 110]. In other tumors, including colorectal cancer (CRC), SIRT6 functions are associated with its tumor suppressive activity [111–113]. Of note, the telomeric protein TRF2 has been newly identified as a novel substrate of SIRT6. Upon exposure to a DNA damaging agent, SIRT6-dependent lysine deacetylation of TRF2 leads to the ubiquitin-dependent proteolysis of the shelterin protein, resulting in downstream proper activation of DDR machinery [114]. An inverse correlation between SIRT6 and TRF2 protein expression levels have been also found in a cohort of CRC patients [114], suggesting that an impairment of TRF2 degradation, as a consequence of SIRT6 loss, could be one of the mechanisms underlying the increased dosages of TRF2 observed in many human malignancies. Whether SIRT6 could also impact on the binding affinity to DNA of TRF2 (and eventually of other shelterin factors) through histone deacetylation remains to be fully elucidated.

### ATRX/DAXX mutations in ALT tumors

Several immortalized cell lines and 10–15% of tumors are telomerase-negative and maintain functional telomeres by utilizing an ALT mechanism (for a review, see refs. [10, 115, 116]). ALT activity has been detected prevalently in cancers from mesenchymal tissues such as bone, soft tissues, neuroendocrine systems, peripheral and central nervous systems [12, 117]. ALT cells show several unusual features, such as highly heterogeneous telomere length [118]. Other markers for ALT include abundant extra-chromosomal double-stranded telomeric DNA prevalently in circular form (t-circles), partially single-stranded telomeric C-rich circles (C-circles), high telomere-specific DDR, telomere sister chromatid exchanges (tSCEs) and formation of APBs (ALT-associated promyelocytic leukemia (PML) nuclear bodies), containing chromosomal or extra-chromosomal telomeric DNA, telomere-associated proteins, and proteins involved in homologous recombination (reviewed in [10]). Several evidences indicate that ALT maintenance is based on DNA recombination [10, 115]. For example, a DNA tag inserted in a single telomere was copied to different telomeres in human ALT cells, but not in telomerase-positive cells [119]. Since HR at telomeres is repressed in normal cells and in telomerase-positive immortalized cells, ALT activation likely requires the inactivation of factors repressing HR. The protein ATRX (a chromatin remodeler of the SWI/SNF family) not only does inhibit HR, but is also able to repress ALT activity if transiently expressed in ALT-positive/ATRX-negative cells [120]. ATRX also binds telomeric repeats and G-quadruplex structures in vitro [121], suggesting that it might play a role in resolving G-quadruplex structures forming at telomeres during replication, thus inhibiting replication fork stalling. Through its ADD domain, ATRX interacts with H3K9me3 [122] and its localization at telomeres is antagonized by TERRA [51]. TERRA also plays a role in ALT that remains to be fully defined. In ALT cancer cells, TERRA levels are higher than in telomerase-positive cancer cells and TERRA transcripts constitutively associate with telomeres [123]. Moreover, a recent finding shows that TERRA directs the enrichment of HP1, H3K9me3, H3K27me3, H4K20me3 in the ALT cell line U2OS, through the recruitment of Polycomb repressive complex 2 (PRC2) [52], typical of facultative heterochromatin. Importantly, at chromosome ends TERRA molecules form RNA-DNA hybrids (R-loops), three-stranded nucleic acid structures consisting of a DNA:RNA hybrid and a displaced DNA strand. The displaced G-rich DNA strand is thought to form G-quadruplex structures, which may cause stalling of replication and DNA damage at telomeres [124], thus increasing homologous recombination among telomeres [125]. Suppression of R-loop formation is one of the multiple functions of ATRX [124], consistent with its ALT suppressing role. However, the main role of ATRX is the deposition - together with the histone chaperone DAXX - of the histone variant H3.3 at pericentric heterochromatic regions and at telomeres [42, 45]. At the moment, it is unknown which role H3.3 deposition at telomeres plays in the development of ALT pathway. However, the importance of the ALT/DAXX/H3.3 pathway is supported by recent surveys of ALT-positive tumors showing a high frequency of mutations in ATRX/DAXX and/or H3.3 [92–95].

### H3.3 mutations in pediatric tumors

Recent studies reported high frequencies of H3.3 mutations in pediatric cancers, often associated with ALT (for a review see [91, 126]). Three residues are involved, respectively Lys27, Gly34, and Lys36. Mutated H3.3K27M (from Lysine to Methionine) and H3.3G34R/V (from Glycine to Arginine or Valine) are frequent in pediatric high-grade glioma (pHGG) or in diffuse intrinsic pontine gliomas (DIPG) [94, 127, 128]. Other two mutations, H3.3K36M and H3.3G34W/L (from Glycine to Tryptophan or Leucine), have been found at high frequency in two juvenile bone tumors, chondroblastomas and giant cell tumors of the bone (GCTBs) [129]. A rare mutation, H3.3K27I (from Lysine to Isoleucine) has been also described in DIPG [130]; moreover, K27M mutation can affect also the canonical histones H3.1 and H3.2 [127, 128, 130]. Although both genes express the same protein product, mutations occur either in *H3F3A* or in *H3F3B* gene. Mutations regarding residues K27 and G34 affect preferentially *H3F3A* gene, whereas K36M mutations occur mostly in *H3F3B* [91]. These missense mutations act in heterozygosis, indicating a “gain of function” role of the mutated histone in cancer development. Remarkably, mutant histones - termed as “oncohistones” [91] due to their dominant nature - are found in pediatric and juvenile tumors but rarely in their adult counterparts. Another peculiar feature is that the anatomical location, the average age at diagnosis, and the overall survival are highly mutation-specific [127, 128, 131]. H3.3G34R/V cancers are found almost exclusively in the cerebral hemispheres, accounting for 16.2% of total cases, and show a longer overall survival compared with other H3.3 mutant groups (median 18 months). H3.1/H3.2 K27M are restricted to the pons (21.4%) and show a median survival of 15 months. H3.3K27M mutations are abundant in the midline and pons, accounting for 63.0% DIPG and 59.7% non-brainstem midline tumors. This group is characterized by a shorter overall survival (median 11 months). The reason for these specificities and the molecular mechanisms at the basis of oncohistones are mostly unknown. The amino acids that are mutated in tumors are sites of possible methylation or acetylation (K27 and K36), or can interfere with post-translational modifications of close lysines (G34). However, the most striking feature of oncohistones is that they act globally, despite the fact that they are expressed by a single allele. Pediatric glioblastomas harboring H3.3K27M mutation show a global reduction of H3K27me3 [132–134]; to a lesser extent, also K27I reduces the global levels of H3K27me3 [132]. Trimethylation of H3K27 is a mark of facultative heterochromatin, catalyzed by PRC2 [135, 136]. In vitro analysis of PRC2 methyltransferase activity and crystal structure studies show that H3K27M inhibits K27 methylation through specific binding to EZH2, the enzymatic subunit of PRC2 [132, 137], leading to a general reprogramming of H3K27me3 and EZH2 on the genome [138]. Recent data suggests that in vivo H3K27M does not bind or sequester PRC2 but instead forms heterotypic H3K27M-K27 ac nucleosomes that interact with bromodomain proteins [139]; in agreement with these results, a recent study shows no increased Ezh2 affinity for nucleosomes containing H3K27M [140].

Similarly to H3K27M mutations, H3.3K36M expression in chondroblastoma correlates with global reduction in H3K36 methylation [141], due to inhibition of NSD2/MMSET, a methyltransferase that catalyzes mono- and di-methylation of H3K36, and SETD2, which catalyzes trimethylation of H3K36me2 [141, 142]. Analogously to H3K36M, it has been proposed that H3.3K36M might act by sequestering NSD2 and SETD2; support to this hypothesis comes from the crystal structure showing a strong binding of H3K36M to the catalytic site of SET2D [143, 144].

The last H3 residue mutated in a subset of pediatric cancers, H3.3G34, is not a site for post-translational modifications, but is in close proximity of H3K36. Indeed, structural analysis showed that H3.3G34R/V/D mutations result in a steric hindrance to the catalytic activity of SETD2 on H3K36 [145]. As a consequence, H3K36 methylation is inhibited also by mutations of H3.3G34 [132, 146], but only in *cis* on the mutant nucleosomes, whereas nucleosomes containing wild-type H3 are not affected by the mutations [132, 146]. Very recently, it has been shown that targeted G34R mutations on one allele of *H3f3a* in mouse embryonic stem (ES) cells resulted in a global epigenetic change [147], namely the inhibition of the KDM4 family of histone demethylases, which target H3 residues K27 and K36. Further analyses are necessary to assess the importance of KDM4 demethylases inhibition in H3.3G34R/V tumors.

## Therapeutic strategies

Therapeutic strategies targeting chromatin modifications are defined as epigenetic therapy. Currently, epigenetic therapy has been proven to be a successful approach for the treatment of hematological malignancies, but little success has been achieved in the treatment of solid tumors (for a recent review see [148]). However, accumulating data on the role of epigenetic alterations occurring at telomeres of cancer cells provides an intriguing and challenging chance for potential targeted therapeutic interventions.

The essential dependence of cancer cells on a telomere maintenance mechanism for replicative immortalization led researchers to investigate these mechanisms as potential cancer-specific therapeutic targets. Given the majority of carcinomas and soft tissue cancers present telomerase activity, whereas telomerase is absent in most normal tissues [11, 149], several efforts have been made to inhibit telomerase by pursuing different strategies: small-molecule inhibitors, antisense oligonucleotides, G-quadruplex stabilizers, immunotherapy, telomerase-driven suicide gene therapy, and chemicals blocking telomerase biogenesis (see ref. [150] for an extensive review). Unfortunately, anti-telomerase approaches have showed effectiveness in only some myeloid tumors but have largely failed in solid tumors (reviewed in [151]). The limitations of targeting telomerase, and the fact that telomerase inhibition would not affect cancer cells using the ALT pathway, encouraged researchers to investigate alternative therapeutic approaches targeting telomeres in a telomerase- and telomere length-independent manner. In agreement with growing findings about the altered telomeric chromatin composition of cancer cells, and considering the pivotal role of shelterin components in telomere protection, targeting telomeric binding factors has been developing as an emerging antitumor approach. Indeed, chemical inhibition of TRF2 [152] or TRF1 [153, 154] were reported to induce rapid DDR activation and growth arrest both in in vitro and in vivo tumor models, respectively.

Until now, telomeric chromatin alterations in cancer have not yet been considered in the design of effective epigenetic therapy, however they can be indirectly targeted by novel identified epigenetic drugs. Due to the broad range of activities and substrates, Sirtuins are involved in several cellular processes, including telomere integrity, but their role in cancer is controversial. These reasons led to the identification of many sirtuin modulators over recent years, both inhibitors and activators, mainly through chemical library screening and catalytic mechanism-based design approaches (reviewed in [155]). Very recently, new chemical activators of SIRT6 have been identified. It has been shown that UBCS039 and MDL-800 are able to inhibit the proliferation of various cell lines regardless of tumor histotype [156–158]. Moreover, MDL-800 compound showed efficacy in a xenograft model of hepatocellular carcinoma [158]. Given the described ability of SIRT6 to affect the protein stability of TRF2 [114], as well as telomere capping, it is reasonable to ask whether the antitumor activities caused by the exposure to SIRT6 activators can be partially attributable to telomere-driven effects. To address this issue, further studies will be needed.

Importantly, there is mounting evidence showing that epigenetic cancer therapy could target ALT-positive gliomas harboring H3.3 mutations [159]. Specifically, recent preclinical studies showed that GSKJ4, a small molecule inhibitor of the histone H3K27 demethylases JMJD3 (KDM6B) and UTX (KDM6A), decreased tumor cell viability and increased H3K27me3 levels in glioma cell lines harboring the mutation of lysine to methionine substitution at codon 27 (K27M), and significantly extended survival of mice with K27M mutant glioma xenografts [160]. In contrast, GSKJ4 has not shown activity in an H3.3G34V mutant glioma cell line [160]. Panobinostat, a histone deacetylase inhibitor, resulted in decreased tumor cell viability in both K27M mutant glioma cell lines and in mice with K27M mutant glioma xenografts [161, 162]. Panobinostat treatment demonstrated a dose dependent increase in histone acetylation and in H3K27me3 [161, 162]. Combined use of GSKJ4 and panobinostat produced a synergistic reduction of tumor cell viability in K27M mutant glioma cell lines [161]. Other strategies to modulate histone methylation are under study, such as targeting EZH2, the histone demethylases KDM1 and KDM5 (see refs. [91, 126, 163] for a review). Strategies that modulate DNA methylation at subtelomeres in ALT are expected to affect cell survival of ALT cells. Additionally, inhibitors of the protein kinase ATR, a regulator of homologous recombination with prolonged recruitment to telomere ends in the setting of ATRX mutation, have been found to selectively induce death of ALT-positive cancer cells [123].

## Conclusions

Telomeres and telomerase have become a main target in developing anticancer strategies, due to their crucial role in cancer development. Many efforts have been focused on telomerase inhibition, however this strategy has therapeutic limits. New anticancer targets could emerge from a clearer comprehension of telomere structure and dynamics. Several aspects of telomere biology need a deeper investigation: the epigenetic pattern of human telomeres is still controversial [38], the role played by the histone H3.3 at telomeres is largely unknown, how telomeric chromatin changes during neoplastic transformation is an issue mostly unexplored.

Effective anticancer strategies require an accurate mapping of the mutations causing the disease, with the ultimate goal to precisely tailor the therapy to the patient. Besides genetic mutations, it is now generally recognized that epigenetic changes play an important role in cancer development [164, 165]. Even if still poorly defined, strategies directed against epigenetic targets have features that can potentially complement classical anticancer approaches, like the possibility to address different pathways at the same time. Characterizing the telomeric epigenome is therefore an important issue, both for a deeper understanding of the telomere protective structure and because it might lead to the emergence of new anti-cancer targets.

## Abbreviations

ALT: Alternative lengthening of telomeres
APB: ALT-associated promyelocitic leukemia (PML) nuclear body
ATRX: α-thalassemia/mental retardation syndrome X-linked
ChIP-seq: Chromatin immunoprecipitation followed by next-generation sequencing
CLL: Chronic lymphocytic leukemia
CRF: Chromatin remodeling factors
DAXX: Death domain-associated protein 6
DDR: DNA damage response
DIPG: Diffuse intrinsic pontine glioma
DSB: Double-strand DNA break
GCTB: Giant cell tumors of the bone
HCC: Hepatocellular carcinoma
HP1: Heterochromatin protein 1
HR: Homologous recombination
iPSC: Induced pluripotent stem cells
ITS: Interstitial telomeric sequences
NHEJ: Non-homologous end-joining
PDGFRβ: Platelet-derived growth factor receptor-β
pHGG: Pediatric high-grade glioma
PML: Promyelocytic leukaemia
TERRA: Telomeric repeat-containing RNA
TPE: Telomere-position effect
TRF1: Telomeric repeat-binding factor 1
TRF2: Telomeric repeat-binding factor 2
tSCE: Telomere sister chromatid exchange
WRN: Werner syndrome ATP-dependent helicase
